# Iatrogenic Teratoma Rupture during TVOR Complicated with Peritonitis, Pleuritis, and Septic Shock

**DOI:** 10.1155/2018/3126436

**Published:** 2018-09-10

**Authors:** Pei-Yi Wang, Yi-En Chang, Yu-Chieh Lee, Chii Ruey Tzeng

**Affiliations:** Department of Obstetrics and Gynecology, Taipei Medical University Hospital, Taipei, Taiwan

## Abstract

**Objective:**

To obtain a better understanding of the clinical course and the subsequent complications of teratoma rupture.

**Case:**

We report a rare case of chemical peritonitis and pleuritis caused by teratoma rupture during ultrasonographically guided transvaginal oocyte retrieval (TVOR). The patient initially presented with nonspecific and digestive symptoms after TVOR, but the condition deteriorated rapidly after three weeks with peritonitis and septic shock. Thus, exploratory laparoscopy was performed with the findings of a ruptured teratoma at left adnexa, severe adhesions, and purulent fluid in her peritoneal cavity. Bilateral pleuritis was also noted after the operation, which was suspected to be caused by chemical irritation of the spilled contents of the teratoma. The patient's condition improved after surgical treatment and was discharged 28 days after admission.

**Conclusion:**

Our case showed that the timing of peritoneal irritation caused by teratoma rupture converting to severe chemical peritonitis was approximately 3 weeks. Physicians should avoid cyst puncture during TVOR and closely observe or even perform surgical treatment when iatrogenic teratoma ruptures are suspected.

## 1. Introduction

Benign cystic teratomas are among the most common ovarian tumors, with a reported incidence of approximately 10%–20% among all ovarian cystic lesions [1]. They commonly occur in women in the reproductive age group and are sometimes complicated by rupture, torsion, or malignant transformation [2]. The most frequently observed symptom is lower abdominal pain (incidence rate: 44.1%–47.6%). These teratomas are often discovered incidentally during other examinations, surgery, or procedures [2, 3].

Rupture accounts for 1%–2% of all complications associated with teratoma. Teratoma rupture can be classified as acute or chronic type. In the chronic type, the leakage of cystic content is slow, and the spillage of sebaceous material may lead to chemical granulomatous peritonitis [4]. The clinical findings of chemical granulomatous peritonitis often mimic those of advanced-stage ovarian carcinomas [5], with intra-abdominal adhesions or masses as frequent sequelae [6]. The causes of teratoma rupture are frequently indeterminate, and spontaneous ruptures are reported in most cases [4]; therefore, a definitive clinical course is often unclear to physicians.

Here, we report a rare case of chemical peritonitis and pleuritis caused by teratoma rupture during ultrasonographically guided transvaginal oocyte retrieval (TVOR). With the definite time of occurrence of the event, we have a better understanding of the clinical course and the subsequent complications of teratoma rupture. Thereby, our experience can serve as a guide for clinical physicians to plan relatively accurate follow-up examinations and treatments if iatrogenic cyst rupture is suspected.

## 2. Case Report

A 42-year-old woman was admitted to our ward from the emergency room (ER), complaining of persistent gastrointestinal discomfort for nearly 3 weeks; she also had fever and severe lower abdominal pain.

The patient did not have any underlying diseases or specific past history, except for infertility and having recently received in vitro fertilization (IVF) treatment. The patient had undergone transvaginal sonography before receiving IVF treatment. Sonography revealed a teratoma-like ovarian mass on the left side, sized approximately 6 × 4 × 4 cm^3^ and several uterine myomas. The IVF-embryo transfer program included the use of a gonadotropin-releasing hormone antagonist, and ovulation was induced using one dose of recombinant human chorionic gonadotropin (250 *µ*g). After 35 hours, she underwent ultrasonographically guided TVOR without any apparent complications and frozen embryo transfer was performed 3 days later. However, pregnancy test 2 weeks later was negative, and patient's failure to conceive in this cycle of treatment was confirmed.

**Figure 1:**
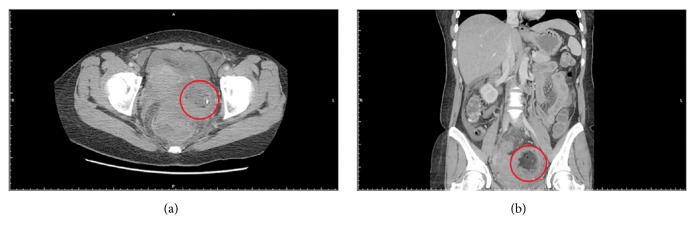
**Abdominal CT survey.** CT arranged in ER showed a mixed density mass about 4*∗*4(cm) with calcifications but discontinuous border over the left adnexa ((a) axial view of abdominal CT; (b) coronal view of abdominal CT).

After undergoing TVOR, the patient experienced dull lower abdominal pain, which persisted for 3 weeks before she decided to visit the ER. Moreover, other symptoms, including fever up to 38°C, severe lower abdominal pain, and watery diarrhea several times a day, had developed 3 days prior to her arrival. The following parameters were recorded upon her arrival at the ER:

- Vital signs: body temperature, 38.5°C; pulse rate, 84 bpm; respiratory rate, 16 bpm, blood pressure, 92/51 mmHg.
- Physical examination: abdominal distension and diffuse lower abdominal tenderness, particularly on the left side.
- Laboratory data: elevated C-reactive protein (CRP) level (11.32 mg/dL), without other specific abnormalities.
- Sonography: cyst and abscess formation on both sides of pelvis with fluid accumulation.
- Computed tomography (to exclude other related diseases): a mixed-density mass, sized approximately 4 × 4 (cm), with calcifications but with a discontinuous border over the left adnexa, and diffused abdominal ascites with mesentery edema; the findings were compatible with clinical suspicion of a ruptured teratoma caused by puncture (of a teratoma) during TVOR (Figure 1).
- Chest X-ray: no specific findings (e.g., no pleural effusion)

Under the impression of peritonitis due to ruptured teratoma, the patient was admitted for further examination and surgical treatment.

Unfortunately, her condition deteriorated rapidly 1 day after admission, with unstable vital signs: pulse rate, 108 bpm; respiratory rate, 46 bpm; blood pressure, 80/57 mmHg. Her urine output also decreased. Physical examination demonstrated diffuse abdominal tenderness, and sonography revealed results similar to those of the previous day. The laboratory data showed some abnormalities, including leukocytosis with WBC count of 34440/*µ*L, neutrophil fraction of 95.4%, CRP level of 34.37 mg/dL, and creatinine level of 1.2 *µ*g/dL. Chest X-ray reports before and after presentation at the ER did not differ considerably. Severe sepsis and accompanied acute kidney injury was initially suspected. We changed the antibiotics from cefazolin + gentamycin + clindamycin to flomoxef + metronidazole, immediately followed by surgical treatment.

**Figure 2:**
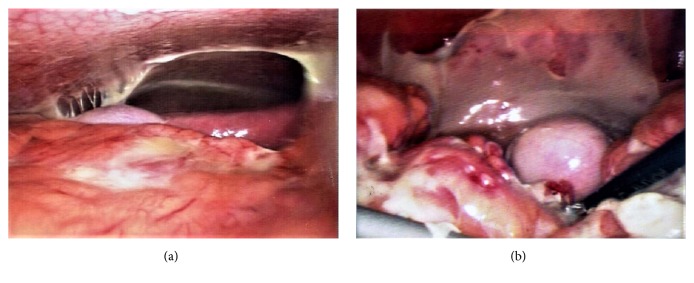
**Operation findings. **(a) Spilled sebaceous content of the ruptured teratoma was seen in peritoneal cavity. (b) A ruptured left teratoma with spread content was noted, which has formed an adhesional complex with the bilateral adnexa. Besides, there was severe adhesion and purulent fluid with the content clotting over the organs in the pelvis.

**Figure 3:**
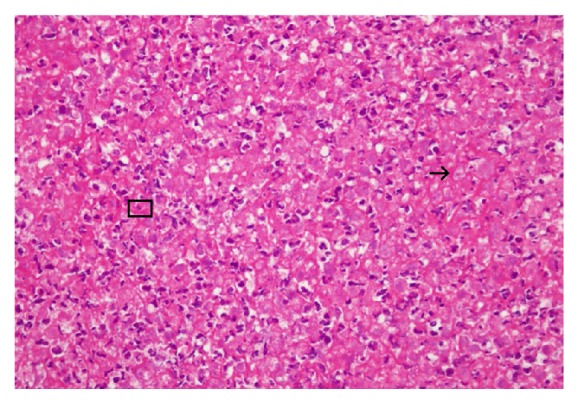
**Pathological studies.** Specimen microscopically showed fibrinopurulent exudate composed of fibrin (the pink homologous background) (arrow), neutrophils, mononuclear inflammatory cells, and cell debris (square).

The patient received exploratory laparoscopy later that day. Upon entering her abdomen, severe adhesions as well as the presence of purulent fluid were found in her peritoneal cavity. Near the left adnexa, a ruptured teratoma and its spilled contents, including sebaceous substances, were noted (Figure 2). Because of severe adhesions caused by active inflammation, it was very difficult to even visualize or to remove the ruptured cyst. Left oophorectomy was also considered at the time but ultimately not performed because of the extent of peritonitis and adhesions. We did, however, remove as much tissue, adhesions, and purulent fluid adjacent to the left adnexa as possible. We also performed washing cytology, arranged intra-abdominal lavage with antibiotics, and inserted bilateral drains. Pathological studies showed that the fibrinopurulent exudate was composed of fibrin, neutrophils, mononuclear inflammatory cells, and cell debris, but without any ovarian tissues (Figure 3).

After the surgery, patient complained of occasional shortness of breath. Bilateral pleuritis was noted on chest X-ray. Drainage with pigtail insertion was subsequently arranged. Cytological findings included an increased number of neutrophils, histiocytes, inflammatory cells, and reactive mesothelial cells on the fibrinoid as well as a bloody background on both sides, which was suspected to be the exudate fluid caused by the chemical irritation of the spilled contents of the teratoma. For the next 2 weeks, patient continued to have intermittent fever, mild abdominal pain, and sporadic shortness of breath, which explained her long stay at the hospital. We continued to administer antibiotics and made the necessary adjustment according to the result of the pus/blood culture (*Escherichia coli* growth was observed in her pus culture, but not in her blood culture). Eventually, patient's condition improved steadily, and she was discharged 28 days after admission.

## 3. Discussion

Although benign cystic teratomas is a common ovarian tumor, in most cases of teratoma ruptures, the causes are frequently indeterminate and reported as spontaneous ruptures. Due to the fact that ruptures often occur without the knowledge of the patients or the doctors, a definitive clinical course remains somewhat unclear. In the case described above, we were able to pinpoint the beginning of the course and thus have a general idea of how teratoma cyst rupture, if left untreated long enough, could evolve clinically. At first, patient presented with symptoms of worsening gastrointestinal tract discomfort. This is similar to previous reports, in which patients with chronic peritonitis caused by the teratoma ruptures also initially presented with nonspecific abdominal and digestive symptoms [4–8]. During the next 3 weeks, the patient developed intermittent fever, severe abdominal pain, and the symptoms even subsequently led to life-threatening complications. This pattern of clinical course was similar to that of the case of teratoma rupture due to TVOR reported by Coccia et al. [7]. Both of these cases showed that the duration it took for the initial peritoneal irritation to evolve to severe chemical peritonitis and even pleuritis was approximately 3 weeks after the occurrence of teratoma rupture. The result might provide clinical physicians with a better understanding of the clinical course of teratoma rupture, as well as the serious, life-threatening consequences associated with it.

Ultrasonographically guided TVOR is a common technique used in the program of in vitro fertilization. However, certain complications, including cyst rupture, infection, and puncture of blood vessels, continue to occur occasionally. During TVOR of our patient, the aspirated fluid was yellowish clear, which resembled the fluid of a mature ovarian follicle. Since no fatty content was noted in the aspirated fluid, we did not suspect that rupture of the teratoma cyst had occurred. It is also due to this reason that we did not perform a transvaginal ultrasound after TVOR. In retrospect, the rupture could have been caused by accidental needle puncture during the procedure. This case serves as a reminder that accidental cyst punctures do occur during TVOR; however, an adequate presurvey including a thorough sonography before the procedure combining with a better maneuver of the puncturing needle can certainly lower the chance of such occurrence. Additionally, if cyst puncture or rupture is suspected to have occurred during TVOR, transvaginal ultrasound should be performed after the procedure in order to exclude free fluid accumulation in the pelvis.

In our experience, the removal of small, benign ovarian cysts before or after performing TVOR is unnecessary in most cases, as iatrogenic cyst rupture is rare, and the rupture of cysts during TVOR does not cause serious damage. In this patient, we also did not plan on removing the teratoma before her pregnancy, because teratomas seldom have adverse effect during pregnancy. It has also been shown in a prospective study by Caspi et al. that teratomas <6 cm are not expected to grow during pregnancy nor to cause complications in pregnancy and labor [9].

Based on this case, the importance of close follow-up with sonography and laboratory data after detection of teratoma rupture should be emphasized. If required, additional treatment for ruptured teratomas should include removal of both the ruptured cyst and the spilled content. Laparoscopic surgery can be a safe and effective procedure [10–13]; compared to laparotomy, it is associated with a lower incidence of postoperative fever and urinary tract infection, a lower postoperative pain score, a shorter duration of hospitalization, and a lower cost [14]. Due to all of these benefits, we consider laparoscopic surgery the technique of choice for removing residual cystic components and performing peritoneal lavage.

Although the condition of our patient improved gradually after surgical treatment with lavage and drainage, whether or not all of the complications for which she suffered from have any effects on her future fertility remains to be seen. A previous study showed that intraoperative spillage of benign cystic teratomas does not cause long-term infertility [15]. Yet, our patient has developed a more serious sequelae, chronic chemical peritonitis, which could possibly affect pelvic organs and subsequent fertility in the long run. More research and studies in this area should give us a clearer answer.

In conclusion, we reported a case of teratoma rupture with a thorough clinical course, which showed that the time it took for the initial peritoneal irritation caused by teratoma rupture to convert to severe chemical peritonitis and even pleuritis was approximately 3 weeks. Furthermore, clinical physicians should avoid cyst puncture during TVOR and closely observe or even perform surgical treatment when iatrogenic teratoma ruptures are suspected.
